# “Surviving and Thriving”: evidence for cortical GABA stabilization in cognitively-intact oldest-old adults

**DOI:** 10.1038/s41398-025-03302-w

**Published:** 2025-03-13

**Authors:** Mark K. Britton, Greg Jensen, Richard AE Edden, Nicolaas AJ Puts, Sara A. Nolin, Stacy Suzanne Merritt, Roxanne F. Rezaei, Megan Forbes, Keyanni Joy Johnson, Pradyumna K. Bharadwaj, Mary Kathryn Franchetti, David A. Raichlen, Cortney J. Jessup, G. Alex Hishaw, Emily J. Van Etten, Aaron T. Gudmundson, Saipavitra Murali-Manohar, Hannah Cowart, Theodore P. Trouard, David S. Geldmacher, Virginia G. Wadley, Noam Alperin, Bonnie E. Levin, Tatjana Rundek, Kristina M. Visscher, Adam J. Woods, Gene E. Alexander, Ronald A. Cohen, Eric C. Porges

**Affiliations:** 1 Department of Epidemiology, College of Public Health and Health Professions & College of Medicine, University of Florida, Gainesville, FL USA; 2 Center for Cognitive Aging and Memory, Evelyn F. and William L. McKnight Brain Institute, University of Florida, Gainesville, FL USA; 3 Department of Psychology, Reed College, Portland, Oregon, USA; 4 Russell H. Morgan Department of Radiology and Radiological Science, School of Medicine, Johns Hopkins University, Baltimore, MD USA; 5 F.M. Kirby Research Center for Functional Brain Imaging, Kennedy Krieger Institute, Baltimore, MD USA; 6 Department of Forensic and Neurodevelopmental Sciences, Institute of Psychiatry, Psychology, and Neuroscience, King’s College London, London, UK; 7 MRC Centre for Neurodevelopmental Disorders, King’s College London, London, UK; 8 Department of Neurology, College of Medicine, Medical University of South Carolina, Charleston, SC USA; 9 Department of Neurology, Miller School of Medicine, University of Miami, Miami, FL USA; 10 Evelyn F. McKnight Brain Institute, Miami, FL USA; 11 Department of Clinical and Health Psychology, College of Public Health and Health Professions, University of Florida, Gainesville, FL USA; 12 Department of Psychology, College of Science, University of Arizona, Tucson, AZ USA; 13 Evelyn F. McKnight Brain Institute, Tucson, AZ USA; 14 VA Boston Healthcare System, Boston, Massachusetts, USA; 15 Department of Biological Sciences, College of Letters, Arts, and Sciences, University of Southern California, Los Angeles, California, USA; 16 Department of Neurology, College of Medicine, University of Arizona, Tucson, AZ USA; 17 Department of Psychiatry, College of Medicine, University of Arizona, Tucson, AZ USA; 18 Department of Neurobiology, Heersink School of Medicine, University of Alabama at Birmingham, Birmingham, AL USA; 19 Evelyn F. McKnight Brain Institute, Birmingham, AL USA; 20 Department of Biomedical Engineering, College of Engineering, University of Arizona, Tucson, AZ USA; 21 Department of Neurology, Heersink School of Medicine, University of Alabama at Birmingham, Birmingham, AL USA; 22 Heersink School of Medicine, University of Alabama at Birmingham, Birmingham, AL USA; 23 Department of Radiology, Miller School of Medicine, University of Miami, Miami, FL USA; 24 Department of Psychology, School of Behavioral and Brain Sciences, University of Texas at Dallas, Dallas, TX USA; 25 Department of Neuroscience, School of Behavioral and Brain Sciences, University of Texas at Dallas, Dallas, TX USA

**Keywords:** Neuroscience, Human behaviour

## Abstract

Age-related alterations in GABAergic function, including depletion of cortical GABA concentrations, is likely associated with declining cognitive performance in normative aging. However, the extent to which GABAergic function is perturbed in the highest-functioning stratum of the oldest-old (85+) population is unknown. For the first time, we report the stability of cortical GABA in this population. We extend our previously-reported Individual Participant Data Meta-Analysis of GABA levels across the lifespan, integrating four large cross-sectional datasets sampling cognitively-intact oldest-old adults. Within our lifespan model, the slope of age-related GABA differences in cognitively-intact oldest-old adults flattens after roughly age 80; within oldest-old adults only, inclusion of age does not improve the fit of models predicting GABA. We interpret these findings as an effect of survivorship: inclusion in the study required intact cognition, and too great a reduction of GABA levels may not be compatible with neurophysiological function needed for intact cognition. This work contributes to a growing body of evidence suggesting that successful cognitive aging may require intact GABAergic function, as well as further characterizing successful aging amongst oldest-old adults and emphasizing GABA as a potential target for interventions to prolong cognitive health in aging.

## Introduction

Gamma-aminobutyric acid (GABA) is the principal inhibitory neurotransmitter in the mammalian brain. In the healthy brain, GABAergic inhibition fine-tunes local and long-range network oscillatory activity in response to external and internal stimuli [1, 2]. On a behavioral level, GABA-mediated cortical inhibitory activity is implicated in a range of cognitive processes, and GABA levels have been associated with cognitive performances across domains, including learning and memory [3], executive function/working memory [4], sensory perception [5], and motor performance [6]. The observed association between GABA and cognition may be due to increased spontaneous neuronal activity and reduced signal-to-noise ratio [7]. Insufficient GABA-mediated inhibitory function is further implicated in uncontrolled excitatory activity and subsequent excitotoxic neuronal death [8]. Consequently, dysfunction in the GABAergic system has been linked to a range of disorders, including epilepsy [9], schizophrenia [10], Major Depressive Disorder [11], Autism Spectrum Disorder [12], and potentially Alzheimer’s disease [13]; it is therefore critical to characterize physiological thresholds at which GABAergic dysregulation may occur.

GABAergic system function is altered in normative aging [14, 15]. Animal models and postmortem human studies indicate that levels of glutamate decarboxylase (GAD), which synthesizes GABA from glutamate, decrease with age [16–18]. GABA receptor subunit expression and function are altered in a region-dependent manner in rodents [19–21]. Additionally, subpopulations of GABAergic interneurons are selectively depleted in rats and humans [22–24] and synaptic contacts decrease [25]. Ultimately, functional capacity for neuronal inhibition is altered, potentially leading to dysregulation of the excitatory-inhibitory balance [15]. Cross-sectional in vivo proton magnetic resonance spectroscopy (MRS) studies in humans have generally reported lower GABA in older adults [26–29], although this finding is not universal [30] and age-related changes in GABA may be regionally-specific [31]. A recent individual participant data meta-analysis by our group demonstrated a gradual and nonlinear slope of age-related cortical GABA differences [32]. Most recently, a within-person analysis demonstrated longitudinal decline in GABA with age [33].

Age-related differences in GABA and associated dysfunctional neuronal inhibition may partially underlie cognitive change in aging. Rodent models suggest that altered excitatory-inhibitory balance and GABAergic system dysfunction may drive declining plasticity in response to task demands [34]. In cross-sectional human MRS studies, lower GABA levels are associated with worse global cognition, fluid processing, sensorimotor performance, and memory performance in normative aging [27, 35–39]. Thus MRS-quantified GABA concentrations may be a meaningful marker for functional capacity in aging, reflecting contributions from intracellular, vesicular, and synaptic GABA pools [40]. Furthermore, inhibitory signaling has been proposed as a target for treatments addressing normative age-related cognitive changes [14], as well as dysfunctional cognitive aging and mild cognitive impairment (MCI) [14].

However, few studies of GABA in normative human aging have included oldest-old adults (85 years and older). Of studies including this population [27, 35], none have examined differences between young-old and oldest-old adults—however, the oldest-old are an atypical population and may be of special interest in researching age-related brain dysfunction. The oldest-old may be disproportionately likely to carry longevity-related physiological traits or engage in protective lifestyles relative to their less long-lived birth cohort peers [41, 42]. In particular, cognitively-intact “successful agers” may be especially resistant to neuropathological processes or able to compensate for neuropathology through sufficiently great cognitive or brain reserve [43]. Given the association between GABA and cognition in normative aging, maintained GABAergic function above an unknown protective threshold may be protective in the cognitively-intact oldest-old (e.g., by reducing excitotoxic injury or maintaining plasticity in networks underlying sensorimotor or learning/memory performance). Consequently, descriptive studies of cognitively-intact oldest-old adults may inform interventions to prolong cognitive health in the general population, as well as in clinical populations affected by GABAergic system dysfunction.

We describe GABA levels among cognitively-intact oldest-old adults, incorporating four novel datasets into a previously-reported individual participant data meta-analysis of GABA across the lifespan [32]. Individual participant data meta-analysis permits consistent statistical modeling across studies, facilitating the use of complex nonlinear models [44]. We report stabilization in the slope of age-related GABA differences among the cognitively-intact oldest-old, potentially related to survivorship in this subpopulation. Within the oldest-old, we report no evidence for age-GABA association (i.e., a flat slope). As exploratory analyses, we additionally examine potential sex differences and GABA-cognition associations within our sample.

## Materials and methods

### Design

206 community-dwelling adults aged 85 to 99 were recruited at the University of Alabama at Birmingham, the University of Florida, the University of Miami, and the University of Arizona as part of a larger study of cognition and brain function in oldest-old adults (McKnight Brain Aging Registry), funded by the Evelyn F. McKnight Brain Foundation. Sites have been designated Site 1-Site 4 to protect participant anonymity. Exclusion criteria were the following: a history of neurological conditions (e.g., MCI, dementia, epilepsy, or major vessel stroke) or intractable psychiatric conditions; active substance use disorder; uncontrolled medical conditions with the potential to limit life expectancy; dependence in activities of daily living or instrumental activities of daily living; less than 6th-grade reading level; and inability to participate in study procedures due to MRI contraindications, vision or hearing deficits, or other major physical disability. Participants were screened for MCI by research coordinators using the Telephone Interview for Cognitive Status-Modified (TICS-M) and Montreal Cognitive Assessment (MoCA): scores of 28 or below on the TICS-M and 22 or below on the MoCA resulted in a consensus conference to determine cognitive status, aided by a neurologist’s evaluation.

### Inclusion

Of the 206 participants recruited, MEGA-PRESS MRS data were acquired from 164 individuals. 39 scans were corrupted or failed visual quality inspection. To ensure that all participants were cognitively-intact, an additional 24 participants scoring 22 or below on the MoCA were excluded from analysis; a cutoff score of 23 has been recommended to minimize the false positive rate for aMCI [45]. An additional participant was excluded due to a GABA+/Cr value falling 4.78 standard deviations from the site mean. A GABA+/Cr fit error criterion of 15% was applied; however, this did not result in the exclusion of additional participants. Demographic data for retained participants (N = 100) are presented in Table 1. Participants self-reported medical history, including history of high blood pressure, cancer treatment, myocardial infarction, and heart failure (Supplementary Table 2).

**Table 1:** Characteristics of McKnight brain aging registry participants retained in final analysis.

Characteristic	Overall, N = 100^a^	Site 1, N = 34^a^	Site 2, N = 18^a^	Site 3, N = 28^a^	Site 4, N = 20^a^
Age	88.66 (3.35)	88.82 (3.62)	89.72 (3.53)	89.07 (3.38)	86.85 (1.87)
Years of Education	16.18 (2.96)	15.59 (2.79)	15.89 (2.47)	17.21 (3.14)	16.00 (3.23)
Sex					
Male	41 (41%)	17 (50%)	8 (44%)	8 (29%)	8 (40%)
Female	59 (59%)	17 (50%)	10 (56%)	20 (71%)	12 (60%)
Race					
White	95 (95%)	32 (94%)	18 (100%)	28 (100%)	17 (85%)
Black	4 (4.0%)	2 (5.9%)	0 (0%)	0 (0%)	2 (10%)
Asian	1 (1.0%)	0 (0%)	0 (0%)	0 (0%)	1 (5.0%)
MoCA Score	25.55 (1.94)	25.26 (1.86)	24.83 (1.98)	26.36 (1.97)	25.55 (1.70)
Voxel Gray Matter Fraction	0.42 (0.05)	0.43 (0.05)	0.43 (0.06)	0.42 (0.05)	0.41 (0.05)

^a^Mean (SD); n (%).

### Imaging

GABA+-edited MEGA-PRESS data were acquired on Siemens 3T scanners (Skyra or Prisma) at each site, using the Siemens MEGA-PRESS WIP. Voxels were 30 × 30 × 30 mm^3^. The voxel was placed on the frontal midline, superior to the genu of the corpus callosum and aligned with the corpus callosum. Chemical shift selective water suppression (CHESS) was utilized [46]. A transversal 20 mm saturation band was placed along the skull, 1–2 mm away from the top of the voxel. Scans were edited for GABA + macromolecules, placing ON editing pulses at 1.9 ppm and OFF at 7.46 ppm; the TE was 68 ms and the TR 2000 ms; 320 averages were acquired (160 ON and 160 OFF). The total scan took 10:48 min. A total of 4096 data points were collected. The spectral width was 4000 Hz. For a subset of 85 participants, unsuppressed water reference scans were separately acquired using the same TE/TR, with water suppression RF pulses deactivated; 32 averages were acquired.

**Fig. 1:**
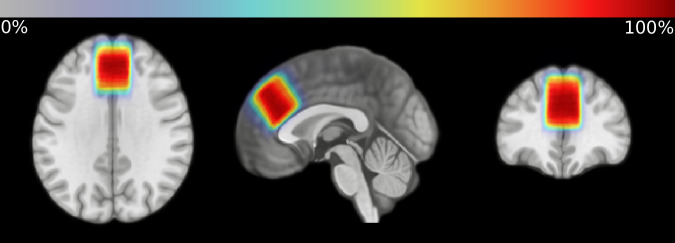
Heatmap of frontal voxel location. The voxel was located superior to the genu of the corpus callosum and aligned with the corpus callosum; voxel location is visualized superimposed over the MNI152 template brain.

**Fig. 2:**
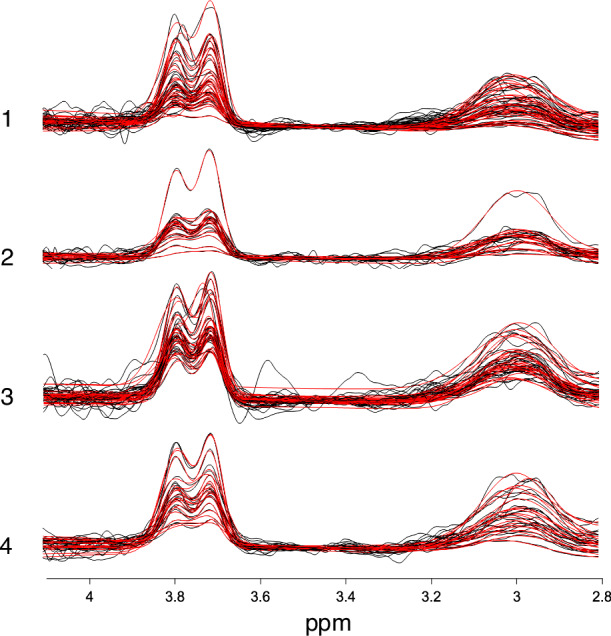
GABA+/Cr and Glx/Cr difference spectra from all sites. The raw difference spectra are plotted in black; the fitted models are plotted in red.

Vendor-native TWIX data were analyzed in Gannet 3.3.1 [47], implemented in MATLAB 2022a. Cr was used as the reference signal; for the 85 participants for whom water reference scans were available, processing was repeated with H2O as the reference signal and the α-correction applied to account for differences in GABA between gray and white matter [48]. No deviations were made from automated procedure. To visualize voxel placement, a heatmap depicting average voxel location normalized to and superimposed over the MNI152 template brain was generated using Osprey 2.5.0 [49] (Fig. 1). Raw difference spectra and fit models by site are shown in Fig. 2. Full details of the analytic pipeline, including GABA SNR, linewidth, and fit error(%), are reported in Supplementary Table 1 per consensus recommendations [50].

### Statistical analysis

All analyses were conducted in R 4.2.2 [51] using the cmdstanr [52] and brms [53] packages to implement Stan 2.32.2 [54]. Following the approach of Porges et al. [32], posterior probability distributions were estimated for all parameters simultaneously using Markov Chain Monte Carlo (MCMC). This approach permits estimates to covary appropriately for non-linear regression.

We first integrated our novel data into our previously-reported lifespan GABA model [32]. Eight other datasets were derived from a systematic literature search identifying studies reporting cortical GABA, acquired using MEGA-PRESS, in healthy populations [26, 27, 35, 55–58]. Only data compatible with an IPD-MA approach were included in the review. A total of eight datasets were included. No data were corrected for voxel tissue fractions. The methods and results of this systematic review have previously been fully reported by our group [32].

Incorporating all 12 datasets, we used cmdstanr to fit a penalized cubic basis spline model [32, 59], which imposes few assumptions on the shape of the age-GABA association over the lifespan. We simultaneously estimated the overall function, a global error term (σ), and one feature scaling factor *F*_*s*_ per dataset. *F*_*s*_ is a posterior probability estimation of the multiplier needed to bring each dataset into standardized units with an overall geometric mean of 1.0 across all cohorts, thereby correcting for systematic methodological differences created by reference method, scanner, voxel location, and unknown site-specific factors in each dataset. This approach assumes that data within a single dataset are comparable, that all data reflect a similar underlying lifespan trajectory, and that age ranges overlap between datasets. We generated *F*_*s*_ as in our prior work [32], weakly penalizing to discourage unlikely extreme values at the tails of the distribution and excluding negative values by setting a lower bound. 19 knots were spaced evenly throughout the model and a smoothing parameter was imposed to minimize overfitting [59]. Estimates of *F*_*s*_ were then compared between water-scaled and Cr-scaled datasets to confirm that methodological similarities would produce more-similar data. To quantify the change in model slope with the addition of the four novel datasets, we took the first derivative of the model, which represents the velocity of GABA+ change across the lifespan.

To assess the impact of age on GABA within cognitively-intact oldest-old adults, we then fit a series of Bayesian linear mixed effects models in brms. Linear models were chosen to minimize the potential impact of overfitting to small variations within a relatively constrained age range (vs. the full lifespan). For all models, 10,000 iterations were run over 4 chains, with a burn-in of 5000. A minimum effective sample size (ESS) of 1000 and $\hat{{\rm{R}}}$ of no greater than 1.05 were considered acceptable evidence of model convergence. Weakly informative priors were set for model fixed effects and residual standard deviation σ (mean = 0.00, SD = 1.00). All continuous variables were centered and scaled within sites.

We initially modeled fixed effects of age and voxel gray matter fraction (fGM) on GABA+/Cr, with a random intercept for site and random slopes for age and gray matter fraction; predictive performance with and without the inclusion of age as a predictor was tested with the leave-one-out (LOO) information criterion [60]. As a supplemental analysis, we fit an additional model of age as a predictor of α-corrected GABA+/H2O in a subset of participants (N = 85). Because stronger age-GABA associations have been reported in women [38], we then repeated our model of GABA+/Cr with sex and an age-by-sex interaction term included as fixed predictors. The LOO information criterion was used to assess predictive performance improvement when including age-by-sex interaction. Finally, to assess variance in cognition explained by GABA within our highly-selected, cognitively-intact study population, we regressed MoCA score on GABA + /Cr, age, and education, using the LOO information criterion to compare predictive performance with and without GABA+/Cr. Random intercepts were again fit by site, and random slopes were fit for GABA+Cr, age, and education.

## Results

**Fig. 3:**
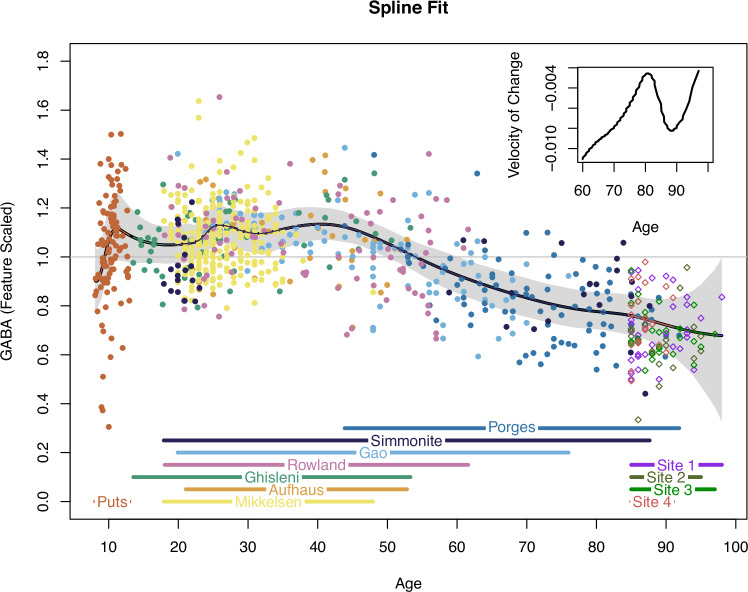
Penalized basis spline model of cortical GABA concentrations across the lifespan and 95% credible interval (shaded). The inset figure depicts a portion of the first derivative of the basis spline model, reflecting the change in steepness of the GABA slope with increasing age.

**Fig. 4:**
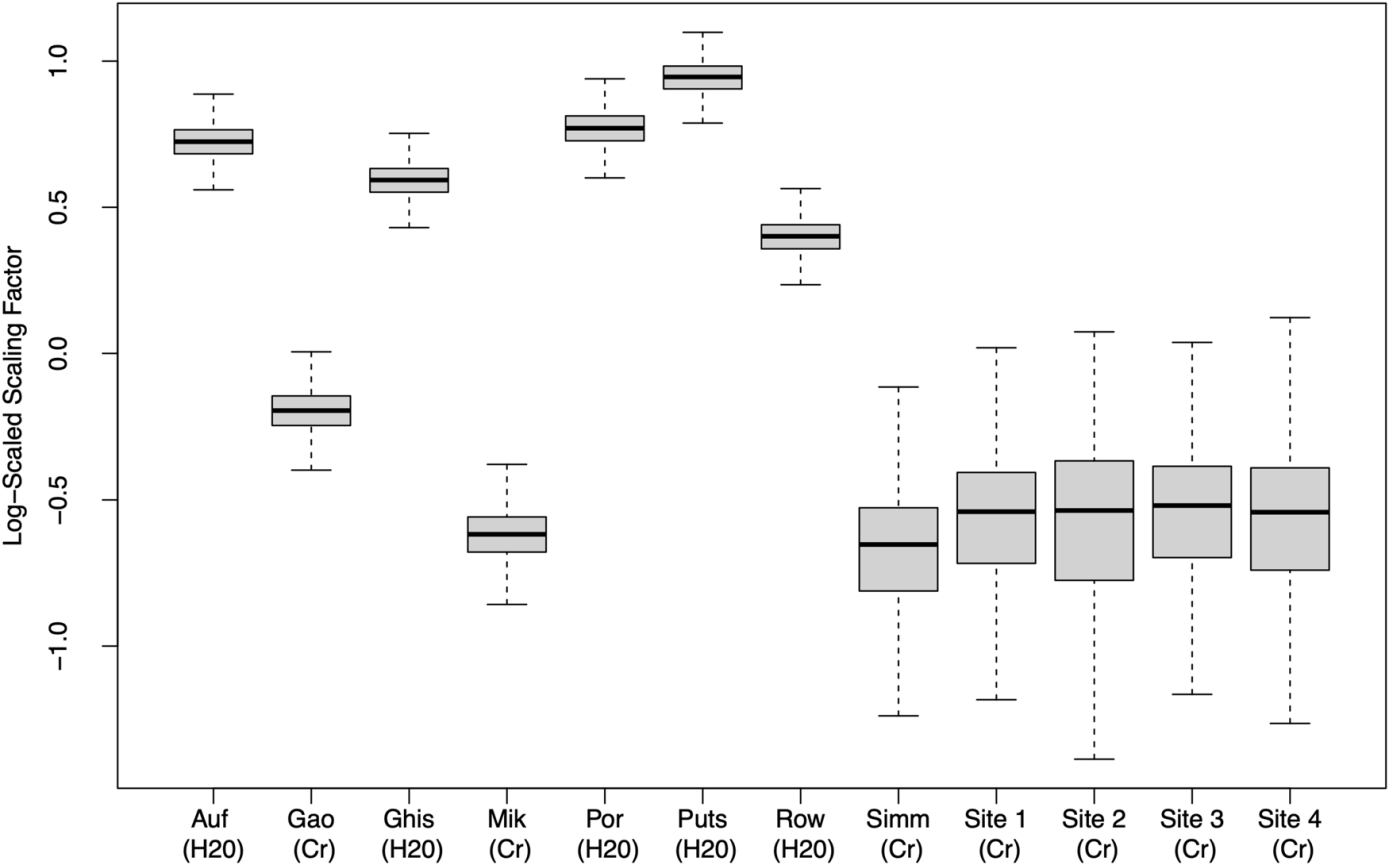
Log-scaled posterior estimates of the feature scaling factor *Fs*. Estimates are shown for each dataset assessed with the spline model.

The mean spline and credible interval from our lifespan nonlinear penalized basis spline model are shown in Fig. 3. The first derivative of the basis spline model after age 60 is inset in Fig. 3. Estimated *F*_*s*_ for each dataset, log-scaled to emphasize ratios and centered to mean *F*_*s*_, is plotted in Fig. 4. Estimates of *F*_*s*_ for the four Cr-referenced novel datasets, like estimates from the three other Cr-referenced datasets (Mikkelsen, Gao, and Simmonite), were smaller than estimates for water-referenced datasets, suggesting consistency between datasets using similar methodologies; this confirms that, although we observed expected site-to-site variability (potentially driven by heterogeneity in imaging protocols [61]) in F_s_, our F_s_-corrected data are likely to be essentially comparable.

The results of our Bayesian mixed-effects model regressing age and voxel gray matter fraction on GABA+/Cr in oldest-old adults are reported in Table 2. LOO information criteria indicated that inclusion of age as a predictor did not clearly improve model predictive performance (elpd_diff_ = −0.5, SE_diff_ = 1.4). The model including age explained 8% of variance (95% CrI = 0.015, 0.17). Posterior predictive plots are provided in Supplementary Fig. 1. Full results of an additional model predicting α-corrected GABA+/H2O are reported in Supplementary Table 3; associations were substantially similar.

**Table 2:** Fixed and random effects and residual standard deviation σ of Bayesian linear mixed effects model of association between age and GABA+/Cr, adjusted for voxel gray matter fraction (fGM).

Parameter	β	Est. Error	95% CrI	$\hat{R}$	Bulk ESS	Tail ESS
Fixed Effects						
Age	0.07	0.22	−0.40, 0.54	1.00	6155	4753
fGM	0.07	0.17	−0.27, 0.41	1.00	7243	4978
Random Effects						
σ_Intercept_	0.18	0.19	0.00, 0.71	1.00	6708	5934
σ_Age_	0.35	0.28	0.02, 1.09	1.00	5324	5348
σ_fGM_	0.20	0.20	0.01, 0.76	1.00	7153	7036
ρ_Intercept, Age_	−0.00	0.51	−0.89, 0.88	1.00	10,216	7991
ρ_Intercept, fGM_	0.01	0.51	−0.89, 0.89	1.00	12,929	8762
ρ_Age,fGM_	0.10	0.50	−0.84, 0.91	1.00	12,654	14,004
Residual Standard Deviation						
σ	1.00	0.08	0.87, 1.16	1.00	17,365	12,983

R^2^ = 0.08 (95% CrI = 0.015, 0.17).

**Fig. 5:**
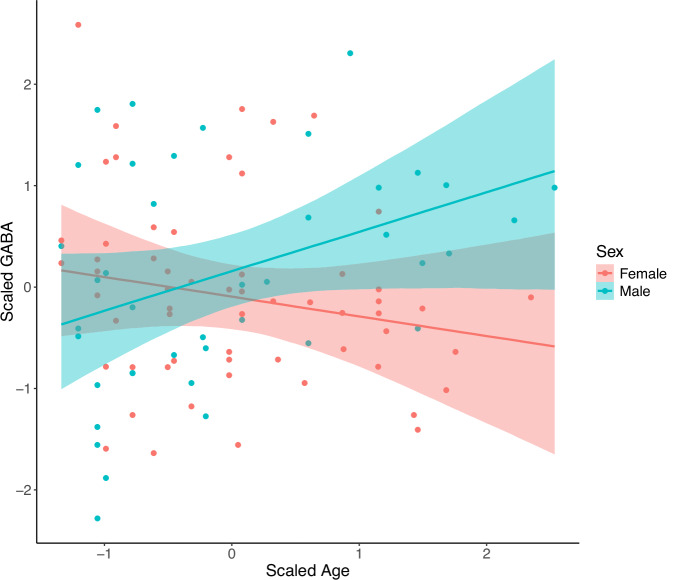
Conditional effect of age by sex on cortical GABA concentrations in oldest-old adults, showing a positive association in oldest-old men only. The model is adjusted for voxel gray matter fraction. The 95% credible interval is shaded.

Our model of age-by-sex interaction showed a positive association between age and GABA for oldest-old males (β = 0.58, 95% CrI = 0.19, 0.98; Fig. 5). Comparison of LOO information criteria revealed an estimated elpd_diff_ of −3.9 (SE_diff_ = 2.4) in favor of inclusion of age-by-sex interaction. The overall model explained an estimated 16% of variance (95% CrI: 0.07, 0.27) and is reported in full in Table 3; posterior predictive plots are provided in Supplementary Fig. 2. Regarding our model of age, education, and GABA+/Cr as predictors of MoCA score, comparison of LOO information criteria indicated that modeling GABA+/Cr did not improve predictive performance (elpd_diff_ = −0.9, SE_diff_ = 1.3). Full model results are reported in Supplementary Table 4, and posterior predictive plots are shown in Supplementary Fig. 3.

**Table 3:** Fixed and random effects and residual standard deviation σ of Bayesian linear mixed effects model of sex as a moderator of age- GABA+/Cr association, adjusted for voxel gray matter fraction (fGM).

Parameter	β	Est. Error	95% CrI	$\hat{R}$	Bulk ESS	Tail ESS
Fixed Effects						
Age	−0.18	0.21	−0.60, 0.27	1.00	5625	3400
fGM	0.14	0.16	−0.19, 0.46	1.00	7224	5389
Sex	0.25	0.20	−0.14, 0.64	1.00	13,546	10,644
Age × Sex_male_	0.58	0.20	0.19, 0.98	1.00	14,780	13,398
Random Effects						
σ_Intercept_	0.18	0.19	0.01, 0.71	1.00	6575	7320
σ_Age_	0.27	0.24	0.01, 0.93	1.00	5564	7234
σ_fGM_	0.19	0.20	0.01, 0.71	1.00	7131	6827
ρ_Intercept, Age_	−0.02	0.52	−0.90, 0.89	1.00	11,012	10,786
ρ_Intercept, fGM_	−0.00	0.51	−0.89, 0.89	1.00	12,912	11,281
ρ_Age, fGM_	0.07	0.51	−0.86, 0.91	1.00	11,764	11,031
Residual Standard Deviation						
σ	0.96	0.07	0.83, 1.12	1.00	15,271	12,829

R^2^ = 0.16 (95% CrI = 0.07, 0.27).

## Discussion

Few studies have examined age-related GABA differences in oldest-old adults (85 + ). We report that the slope of age-related differences in cortical GABA may be flat in cognitively-intact oldest-old adults: our nonparametric lifespan model appeared to flatten after age 80. Supporting this finding, the velocity of change in GABA decreases after age 80, apparently reflecting the introduction into the model of a large number of cognitively-intact oldest-old individuals in this age group, and only begins to increase again by 90. Within cognitively-intact oldest-old adults only, predictive performance for GABA was not improved by inclusion of age in our model [62]. However, exploratory analysis within oldest-old adults was consistent with potential sex differences, such that oldest-old males showed a linear increase in GABA with increasing age.

The observed effect may be driven by survivorship and sample selection. The mean MoCA score in our sample was 25.55 (SD = 1.94; range 23–30). In cognitively-normal oldest-old adults, normative means of 25.09 (SD = 3.04) for 80-to-89-year-olds and 23.73 (SD = 3.01) for 90-to-99-year-olds have been reported [63]; conversely, our participants exceeded population-based normative means for non-demented 70 to 80-year-olds by roughly .88 standard deviations, suggesting that our sample performed on par with or outperformed young-old adults in the general population [64].

**Fig. 6:**
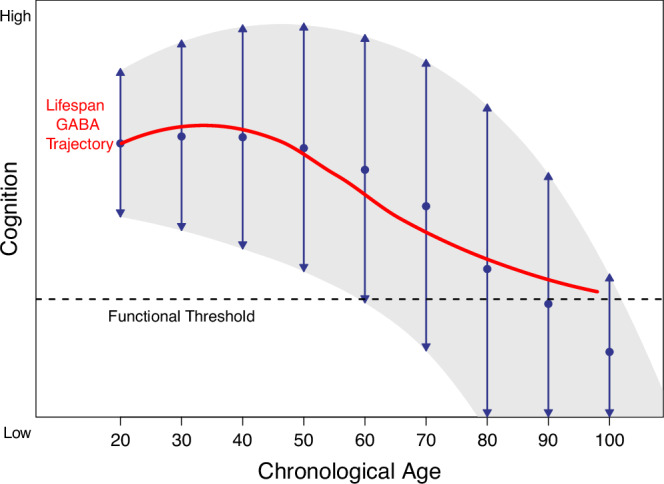
Smoothed GABA spline data are superimposed over a conceptual model of age-related cognitive change. Age-associated differences in GABA may function as a biological influence modulating trajectories of cognition in aging [68]. Figure adapted from Hertzog et al. [68].

Cognitively-intact oldest-old adults are a highly-selected, atypical minority of their birth cohort. Most individuals do not live to 85, and those who do may be physiologically or behaviorally unusual [41, 42]. Even among the minority of adults who reach oldest-old age, the estimated prevalences of MCI and dementia are 18–35% [65, 66] and 16–23% [67], respectively. That is, during typical aging, accumulating physiological changes (in this case, reduced GABA levels) ultimately exceed ranges compatible with normal cognitive function, leading to age-related cognitive decline [68] (Fig. 6). Conversely, the cognitively-intact oldest-old adults in our nonclinical sample exist above this hypothesized minimum GABA “floor” for sustained cognitive function, consistent with the observed attenuated slope of age-related GABA differences. We characterize this as a “surviving and thriving” effect.

Notably, within cognitively-intact oldest-old adults, inclusion of GABA + /Cr in mixed effects models of MoCA score adjusted for age, education, and gray matter fraction did not improve model predictive performance. This finding stands in contrast with prior studies reporting linear GABA-MoCA associations in mixed young-old and oldest-old samples [27]. We interpret this discrepancy as the result of less-dramatic survivorship effects among young-old adults: grossly cognitively-intact young-old adults may be less physiologically atypical than cognitively-intact oldest-old adults and, therefore, may show a greater range of age-related physiological markers. Conversely, in an oldest-old sample selected for atypical preservation of cognitive function, further increases in GABA may show diminishing cognitive returns; once the necessary GABA threshold is exceeded, other aspects of brain function may better predict performance. It should also be noted that there was relatively little variance in MoCA score within our sample.

We observed a positive association between age and GABA in oldest-old men only. Rather than reflecting increased GABA synthesis or reduced GABA breakdown with advancing age in men only, we interpret this cross-sectional association as driven in part by particularly-stringent survivorship effects among aging men. Across demographic groups, men consistently have shorter lifespans [69]; consequently, in cross-sectional samples of the surviving oldest-old, men may be more atypical (e.g., lower exposure to environmental or behavioral risk factors for mortality or greater resilience) relative to birth cohort peers than are similarly-aged women. In combination with demographic and epidemiologic evidence for sex differences in risk behavior within birth cohorts (e.g., historically earlier widespread adoption of cigarette smoking among men [70]) and sex differences in aging-related morbidity (e.g., dementia in women [71]), this observation suggests that demographic factors should be considered when assessing survivorship effects in the oldest-old. However, it should be noted that LOO comparison may be less reliable in small samples (n < 100) [72], and this result should be replicated in larger studies.

Our findings, taken in context, are consistent with prior evidence that successful cognitive aging is associated with higher GABA. Extending these findings, we suggest that GABA concentration (compared to birth cohort peers) may be a viable predictive biomarker of future cognitive trajectory, and potentially of morbidity and/or mortality, in oldest-old adults. Additionally, the GABAergic system may be a viable target for interventions to prevent or reverse normative age-related cognitive decline, as well as a potential target in clinical populations (e.g., individuals at risk for dementia) [14]. α5-GABA-A receptor positive allosteric modulators have shown early promise in reversing learning and memory deficits in rodent models [11], with some compounds showing benefit specific to aged animals [73]. Somatotropic supplementation improves cognition in rat and human studies of normative aging and MCI, potentially by attenuating age-related changes in GABAergic inhibitory tone [74, 75]. GABA_B_ receptor antagonists facilitate learning in aged rats [76] and improved executive function and processing speed performance in a Phase II human trial in MCI [77]. These data suggest collectively that targeting GABAergic dysfunction pharmacologically may partially remediate age-related declines in cognitive performance, although further work in humans is needed to characterize long-term safety and efficacy in MCI and normative aging.

To identify targets for intervention, further research is needed to characterize the mechanism or mechanisms driving age-related GABAergic differences. Reduced concentrations of GABA may reflect decreases in GABA synthesis: regional depletion of GAD65 and GAD67 has been reported in rodent models of aging [15], and cortical GAD65 is reduced in older adults in postmortem human studies [18]. However, reductions in GABA concentrations may also potentially reflect age-related changes in GABA recycling and metabolism. Furthermore, MRS does not quantify the presence or activity of GABA receptors or GABAergic interneurons; nor does MRS directly quantify synaptic activity [78, 79]. Animal models are consistent with age-related alterations in receptor expression [19–21], number of synaptic contacts [25], and number of GABAergic interneurons [22–24]; however, less is known about parallel changes in humans. Furthermore, the impact of age on these aspects of GABAergic function is likely region-dependent and may not be purely inhibitory: for instance, age-related changes in GABA_B_ receptor expression may locally upregulate excitation or inhibition, depending on whether the receptor is pre- or post-synaptic [15]. Thus the extent to which receptor expression and synaptic activity are altered or GABAergic interneurons are depleted in cognitively-intact oldest-old adults remains a question for further study. Finally, age-related changes in ambient GABA quantified by MRS may be partially explained by gray matter atrophy during aging [80]. In sum, additional research is needed to link molecular evidence to in vivo GABA studies for a unified understanding of changes in GABA in normative and pathological aging in humans.

Strengths of our analysis include the individual participant data meta-analytic approach, which allows us to leverage datasets covering the full lifespan; additionally, our Markov Chain Monte Carlo approach reduces the impact of inter-dataset methodological heterogeneity on our model. However, our analysis has several limitations. First, although our model represents the full lifespan, all included data are cross-sectional. Therefore, we cannot model individual slopes; some apparently-intact participants may have been masking neuropathology due to high baseline function [81], although these individuals are likely still overall healthier than currently-impaired peers. Second, we did not directly compare our cognitively-intact sample to oldest-old adults with MCI or dementia; we hypothesize that impaired oldest-old adults would show lower GABA levels, consistent with evidence that GABA is lower in older adults with MCI or dementia [82, 83]. Third, we were unable to assess demographic characteristics for our full lifespan model due to unavailability of individual demographic data for the eight previously-published datasets. Furthermore, our sample was predominantly White and well-educated, and associations may differ in other groups. Additionally, potential lifestyle influences on GABA, such as lifetime substance use history, are beyond the scope of the present analysis. Finally, we analyzed only a small number of participants over the age of 95, resulting in greater uncertainty regarding the trajectory of GABA differences after 95 (and a wider credible interval).

In conclusion, we observed attenuation of the slope of age-related differences in cortical GABA concentrations in oldest-old age, consistent with a minimum GABA “floor” physiologically necessary to sustain cognitive function. This lower limit may also reflect a state below which excitotoxic processes accelerate. The current analysis provides a descriptive benchmark for future studies examining GABA change in the oldest-old population, as well as for studies assessing the impact of aging-like pathological processes on GABA-mediated inhibitory function; additionally, this analysis highlights the impact of study inclusion criteria and survivorship on observed physiological stability in oldest-old age.

## Supplementary information

Supplemental Material

## Data Availability

All code and previously-published data, including data reported in our previously-reported IPD-MA model, are publicly accessible from the Open Science Framework [84]. McKnight Brain Aging Registry data are available from the McKnight Brain Research Foundation upon reasonable request.
