# Supplementary material for: “Surviving and Thriving”: evidence for cortical GABA stabilization in cognitively-intact oldest-old adults
Source: Transl Psychiatry. 2025 Mar 13;15:79. doi: 10.1038/s41398-025-03302-w (PMC11906729; doi:10.1038/s41398-025-03302-w)
Supplement: Supplementary file 1 — Supplemental Material [file 41398_2025_3302_MOESM1_ESM.docx]

Supplementary Table 1: Summary MRSinMRS report following MRSinMRS consensus minimum reporting standards.^1^

| 1. Hardware | |
| --- | --- |
| a. Field strength [T] | 3T |
| b. Manufacturer | \| Dataset \| Manufacturer \| \| --- \| --- \| \| Aufhaus \| Siemens \| \| Gao \| Philips \| \| Ghisleni \| GE \| \| Mikkelsen \| GE/Philips/Siemens \| \| Porges \| Philips \| \| Puts \| Philips \| \| Rowland \| Siemens \| \| Simmonite \| Philips \| \| MBAR Site 1 \| Siemens \| \| MBAR Site 2 \| Siemens \| \| MBAR Site 3 \| Siemens \| \| MBAR Site 4 \| Siemens \| |
| c. Model (software version if available) | \| Dataset \| Model \| \| --- \| --- \| \| Aufhaus \| Magnetom TIM Trio \| \| Gao \| Achieva \| \| Ghisleni \| HD.xt \| \| Mikkelsen \| 24-site study; site-specific information available in origin al manuscript^2^ \| \| Porges \| Achieva \| \| Puts \| Achieva \| \| Rowland \| Tim Trio \| \| Simmonite \| Ingenia \| \| MBAR Site 1 \| Magnetom Prisma \| \| MBAR Site 2 \| Magnetom Skyra \| \| MBAR Site 3 \| Magnetom Prisma \| \| MBAR Site 4 \| Magnetom Skyra \| |
| d. RF coils: nuclei (transmit/receive), number of channels, type, body part | \| Dataset \| Coil \| \| --- \| --- \| \| Aufhaus \| 1H 32-channel head coil \| \| Gao \| 1H 8-channel head coil \| \| Ghisleni \| 1H 8-channel head coil \| \| Mikkelsen \| 24-site study; site-specific information available in original manuscript^2^ \| \| Porges \| 1H 32-channel head coil \| \| Puts \| 1H 32-channel head coil \| \| Rowland \| 1H 32-channel head coil \| \| Simmonite \| 1H 32-channel head coil \| \| MBAR Site 1 \| 1H 64-channel head coil \| \| MBAR Site 2 \| 1H 64-channel head coil \| \| MBAR Site 3 \| 1H 64-channel head coil \| \| MBAR Site 4 \| 1H 64-channel head coil \| |
| e. Additional hardware | - |

| 2. Acquisition | |
| --- | --- |
| a. Pulse sequence | MEGA-PRESS (Mescher et al., 1998) |
| b. Volume of interest (VOI) locations | \| Dataset \| Location \| \| --- \| --- \| \| Aufhaus \| Medial frontal \| \| Gao \| Medial frontal \| \| Ghisleni \| Dorsolateral prefrontal \| \| Mikkelsen \| Parietal \| \| Porges \| Medial frontal \| \| Puts \| Right precentral sulcus \| \| Rowland \| Medial frontal \| \| Simmonite \| Medial occipital \| \| MBAR Site 1 \| Medial frontal \| \| MBAR Site 2 \| Medial frontal \| \| MBAR Site 3 \| Medial frontal \| \| MBAR Site 4 \| Medial frontal \| |
| c. Nominal VOI size [mm^3^] | \| Dataset \| Voxel Size \| \| --- \| --- \| \| Aufhaus \| 40 x 30 x 20 mm^3^ \| \| Gao \| 30 x 30 x 30 mm^3^ \| \| Ghisleni \| 25 x 40 x 30 mm^3^ \| \| Mikkelsen \| 30 x 30 x 30 mm^3^ \| \| Porges \| 30 x 30 x 30 mm^3^ \| \| Puts \| 30 x 30 x 30 mm^3^ \| \| Rowland \| 40 x 30 x 20 mm^3^ \| \| Simmonite \| 30 x 30 x 25 mm^3^ \| \| MBAR Site 1 \| 30 x 30 x 30 mm^3^ \| \| MBAR Site 2 \| 30 x 30 x 30 mm^3^ \| \| MBAR Site 3 \| 30 x 30 x 30 mm^3^ \| \| MBAR Site 4 \| 30 x 30 x 30 mm^3^ \| |
| d. Repetition time (TR), echo time (TE) [ms] | \| Dataset \| Parameters \| \| --- \| --- \| \| Aufhaus \| TR 3000ms; TE 68ms \| \| Gao \| TR 2000ms; TE 68ms \| \| Ghisleni \| TR 2000ms; TE 68ms \| \| Mikkelsen \| TR 2000ms; TE 68ms \| \| Porges \| TR 2000ms; TE 68ms \| \| Puts \| TR 2000ms; TE 68ms \| \| Rowland \| TR 2000ms; TE 68ms \| \| Simmonite \| TR 1800ms; TE 68ms \| \| MBAR Site 1 \| TR 2000ms; TE 68ms \| \| MBAR Site 2 \| TR 2000ms; TE 68ms \| \| MBAR Site 3 \| TR 2000ms; TE 68ms \| \| MBAR Site 4 \| TR 2000ms; TE 68ms \| |
| e. Total number of averages per spectrum  i. Number of averaged spectra per subspectrum | \| Dataset \| Averages \| \| --- \| --- \| \| Aufhaus \| 192 total; 96 per subspectrum \| \| Gao \| 320 total; 160 per subspectrum \| \| Ghisleni \| 320 total; 160 per subspectrum \| \| Mikkelsen \| 320 total; 160 per subspectrum \| \| Porges \| 320 total; 160 per subspectrum \| \| Puts \| 320 total; 160 per subspectrum \| \| Rowland \| 256 total; 128 per subspectrum \| \| Simmonite \| 256 total; 128 per subspectrum \| \| MBAR Site 1 \| 320 total; 160 per subspectrum \| \| MBAR Site 2 \| 320 total; 160 per subspectrum \| \| MBAR Site 3 \| 320 total; 160 per subspectrum \| \| MBAR Site 4 \| 320 total; 160 per subspectrum \| |
| f. Additional sequence parameters  i. Editing pulse frequencies | \| Dataset \| Parameters \| \| --- \| --- \| \| Aufhaus \| F1 not reported, 4096 points ppm_ON_ = 1.9 ppm_OFF_ = 7.5 \| \| Gao \| F1: 1000 Hz Points not reported ppm_ON_ = 1.9 ppm_OFF_ = 7.5 \| \| Ghisleni \| F1 and points not reported; cited methods paper reports ppm_ON_: 1.9 and ppm_OFF_: 7.6 \| \| Mikkelsen \| F1 and number of points site-specific^2^ ppm_ON_: 1.9 ppm_OFF_: 7.46 \| \| Porges \| F1: 2000 Hz 2048 points ppm_ON_: 1.9ppm ppm_OFF_: 7.46ppm \| \| Puts \| F1: 2000 Hz 2048 points ppm_ON_ = 1.9 ppm_OFF_ = 7.46 \| \| Rowland \| F1 and points not reported ppm_ON_: 1.9 ppm_OFF_: 1.5 \| \| Simmonite \| F1: 2000 Hz 2000 points ppm_ON_: 1.90 ppm_OFF_: 7.46 \| \| MBAR Site 1 \| F1: 4000 Hz,  4096 points ppm_ON_ = 1.90, ppm_OFF_ = 7.50 \| \| MBAR Site 2 \| F1: 4000 Hz,  4096 points ppm_ON_ = 1.90, ppm_OFF_ = 7.50 \| \| MBAR Site 3 \| F1: 4000 Hz,  4096 points ppm_ON_ = 1.90, ppm_OFF_ = 7.50 \| \| MBAR Site 4 \| F1: 4000 Hz,  4096 points ppm_ON_ = 1.90, ppm_OFF_ = 7.50 \| |
| g. Water suppression method | \| Dataset \| Method \| \| --- \| --- \| \| Aufhaus \| Not reported \| \| Gao \| CHESS \| \| Ghisleni \| Not reported \| \| Mikkelsen \| 24-site study; site-specific information available in original manuscript^2^ \| \| Porges \| VAPOR \| \| Puts \| VAPOR \| \| Rowland \| WET \| \| Simmonite \| VAPOR \| \| MBAR Site 1 \| CHESS \| \| MBAR Site 2 \| CHESS \| \| MBAR Site 3 \| CHESS \| \| MBAR Site 4 \| CHESS \| |
| h. Shimming method, reference peak, and threshold of acceptance of shim chosen | \| Dataset \| Method \| \| --- \| --- \| \| Aufhaus \| Not reported \| \| Gao \| FASTMAP \| \| Ghisleni \| Not reported \| \| Mikkelsen \| 24-site study; site-specific information available in original manuscript^2^ \| \| Porges \| Not reported \| \| Puts \| Not reported \| \| Rowland \| Automated and manual shimming to achieve 12Hz water linewidth \| \| Simmonite \| Not reported \| \| MBAR Site 1 \| Vendor-standard automated shimming \| \| MBAR Site 2 \| Vendor-standard automated shimming \| \| MBAR Site 3 \| Vendor-standard automated shimming \| \| MBAR Site 4 \| Vendor-standard automated shimming \| |
| i. Trigger or motion correction | - |

| 3. Data analysis methods and outputs | |
| --- | --- |
| a. Analysis software | \| Dataset \| Software \| \| --- \| --- \| \| Aufhaus \| jMRUI/LCModel \| \| Gao \| jMRUI 4.0 \| \| Ghisleni \| LCModel 6.3 \| \| Mikkelsen \| Gannet \| \| Porges \| Gannet 2.0 \| \| Puts \| Gannet 2.0 \| \| Rowland \| Gannet 2.0 \| \| Simmonite \| Gannet \| \| MBAR Site 1 \| Gannet 3.3.1 \| \| MBAR Site 2 \| Gannet 3.3.1 \| \| MBAR Site 3 \| Gannet 3.3.1 \| \| MBAR Site 4 \| Gannet 3.3.1 \| |
| b. Processing steps deviating from default | \| Dataset \| Deviations \| \| --- \| --- \| \| Aufhaus \| None \| \| Gao \| None \| \| Ghisleni \| None \| \| Mikkelsen \| Transient pairs greater than 3 SDs from mean of pre-corrected transients removed from analysis; noise was estimated for two detrended segments of the spectrum and the smaller SD used \| \| Porges \| None \| \| Puts \| None \| \| Rowland \| None \| \| Simmonite \| None \| \| MBAR Site 1 \| None \| \| MBAR Site 2 \| None \| \| MBAR Site 3 \| None \| \| MBAR Site 4 \| None \| |
| c. Output measure | \| Dataset \| Measure \| \| --- \| --- \| \| Aufhaus \| GABA/H2O \| \| Gao \| GABA+/Cr \| \| Ghisleni \| GABA+/H2O \| \| Mikkelsen \| GABA+/Cr \| \| Porges \| GABA+/H2O \| \| Puts \| GABA+/H2O \| \| Rowland \| GABA/H2O \| \| Simmonite \| GABA+/Cr \| \| MBAR Site 1 \| GABA+/Cr \| \| MBAR Site 2 \| GABA+/Cr \| \| MBAR Site 3 \| GABA+/Cr \| \| MBAR Site 4 \| GABA+/Cr \| |
| d. Quantification references and assumptions, fitting model assumptions | \| Dataset \| Assumptions \| \| --- \| --- \| \| Aufhaus \| Nonlinear least-squares algorithm fit to GABA+; concentration in 199 mM GABA phantom used to convert *in vivo* GABA to semiquantitative institutional units \| \| Gao \| Nonlinear least-squares (AMARES) algorithm fit to GABA+ \| \| Ghisleni \| Linear combination modeling of GABA+ and other metabolite peaks \| \| Mikkelsen \| Nonlinear least-squares fit to GABAGlx using three-Gaussian function w/ nonlinear baseline \| \| Porges \| Nonlinear least-squares fit to GABAGlx using Gaussian peak \| \| Puts \| Nonlinear least-squares fit to GABAGlx using Gaussian peak \| \| Rowland \| Nonlinear least-squares fit to GABAGlx using Gaussian peak \| \| Simmonite \| Nonlinear least-squares fit to GABAGlx using Gaussian peak \| \| MBAR Site 1 \| Nonlinear least-squares fit to GABAGlx using Gaussian peak w/ linear baseline \| \| MBAR Site 2 \| Nonlinear least-squares fit to GABAGlx using Gaussian peak w/ linear baseline \| \| MBAR Site 3 \| Nonlinear least-squares fit to GABAGlx using Gaussian peak w/ linear baseline \| \| MBAR Site 4 \| Nonlinear least-squares fit to GABAGlx using Gaussian peak w/ linear baseline \| |

| 4. Data quality | |
| --- | --- |
| a. SNR (GABA), linewidth (GABA) [Hz] | \| Site \| SNR \| FWHM \| \| --- \| --- \| --- \| \| Aufhaus \| Not reported \| Not reported \| \| Gao \| Not reported \| Not reported \| \| Ghisleni \| Not reported \| 5.9 (1.0) for young adults; 6.7 (1.1) for older adults \| \| Mikkelsen \| 25(8) \| 8.10(0.83) \| \| Porges \| Not reported \| 18.6(2.38)^[[1]](#footnote-2)^ \| \| Puts \| Not reported \| 18.3 (3.06)^[[2]](#footnote-3)^ \| \| Rowland \| Not reported \| Not reported \| \| Simmonite \| Not reported \| Not reported \| \| Site 1 \| 16.14(3.79) \| 21.33(2.36) \| \| Site 2 \| 14.83(4.41) \| 20.20(3.68) \| \| Site 3 \| 17.33(3.75) \| 21.23(2.08) \| \| Site 4 \| 18.53(5.38) \| 21.25(2.66) \| |
| b. Data exclusion criteria | \| Site \| Criteria \| \| --- \| --- \| \| Aufhaus \| Failure of visual inspection \| \| Gao \| Relative CRSD(%) < 10 \| \| Ghisleni \| Not reported for main analysis \| \| Mikkelsen \| Failure of visual inspection or failure of signal fitting \| \| Porges \| Failure of visual inspection \| \| Puts \| Failure of visual inspection or model failure to converge \| \| Rowland \| Normalized fitting residual(%) > 15 \| \| Simmonite \| CRLB(%) < 20 \| \| Site 1 \| Failure of visual inspection GABA+/Cr > 3 SDs from site mean GABA+/Cr fit error(%) > 15 \| \| Site 2 \| Failure of visual inspection GABA+/Cr > 3 SDs from site mean GABA+/Cr fit error(%) > 15 MoCA < 23 \| \| Site 3 \| Failure of visual inspection GABA+/Cr > 3 SDs from site mean GABA+/Cr fit error(%) > 15 \| \| Site 4 \| Failure of visual inspection GABA+/Cr > 3 SDs from site mean GABA+/Cr fit error(%) > 15 \| |
| c. Quality measures of post-processing model fitting | \| Site \| Measure \| \| --- \| --- \| \| Aufhaus \| Not reported \| \| Gao \| Mean relative CRSD 5.33 \| \| Ghisleni \| CRLB 4.6(0.9) for younger and 4.4(0.9) for older \| \| Mikkelsen \| GABA+ fit error 5.70(1.45) \| \| Porges \| GABA+ fit error 6.21(1.24)^[[3]](#footnote-4)^ \| \| Puts \| GABA+ fit error 5.57(1.69) \| \| Rowland \| Normalized fitting residual 6.7(1.8) \| \| Simmonite \| Not reported \| \| Site 1 \| GABA+ fit error 6.20(1.66) \| \| Site 2 \| GABA+ fit error 7.12(2.98) \| \| Site 3 \| GABA+ fit error 6.30(2.57) \| \| Site 4 \| GABA+ fit error 5.80(1.06) \| |
| d. Visualized spectra and model fits | Figure 2 (MBAR Site 1-4 only) |

Supplementary Table 2: Self-reported medical history of included participants. Due to the high lifetime prevalence of skin cancer in our cohort, reported cancers are divided by primary site (skin/other cancers). Participants could report more than one type of cancer.

| **Characteristic** | **Overall**, N = 100^1^ | **1**, N = 34^1^ | **2**, N = 18^1^ | **3**, N = 28^1^ | **4**, N = 20^1^ |
| --- | --- | --- | --- | --- | --- |
| Cancer | 47 (47%) | 20 (59%) | 6 (33%) | 14 (50%) | 7 (37%) |
| Skin Cancer^2^ | 20/47 (43%) | 9/20 (45%) | 4/6 (67%) | 6/14 (43%) | 1/7 (14%) |
| Other Cancers^3^ | 27/47 (57%) | 11/20 (55%) | 2/6 (33%) | 8/14 (57%) | 6/7 (86%) |
| Missing | 1 | 0 | 0 | 0 | 1 |
| Myocardial Infarction | 4 (4.0%) | 3 (8.8%) | 0 (0%) | 1 (3.6%) | 0 (0%) |
| Missing | 1 | 0 | 0 | 0 | 1 |
| Heart Failure | 1 (1.0%) | 1 (2.9%) | 0 (0%) | 0 (0%) | 0 (0%) |
| Missing | 1 | 0 | 0 | 0 | 1 |
| Coronary Artery Disease | 7 (7.1%) | 2 (5.9%) | 0 (0%) | 3 (11%) | 2 (11%) |
| Missing | 1 | 0 | 0 | 0 | 1 |
| Hypertension | 60 (61%) | 20 (59%) | 11 (61%) | 20 (71%) | 9 (47%) |
| Missing | 1 | 0 | 0 | 0 | 1 |
| ^1^n (%)  ^2^Includes squamous cell carcinoma (N=4), melanoma (N=3), basal cell carcinoma (N=1), and unspecified skin cancer (N=12) ^3^Includes breast cancer (N=8), colorectal cancer (N=6), kidney or ureter cancer (N=2), stomach cancer (N=1), bladder cancer (N=2), prostate cancer(N=7), ovarian cancer (N=1), lung cancer (N=1), and cervical cancer (N=1) | | | | | |

*Supplementary Table 3.* Fixed and random effects and residual standard deviation σ of Bayesian linear mixed effects model of association between age and alpha-corrected GABA+/H2O.

| **Parameter** | **β** | **Est. Error** | **95% CrI** | $\hat{\boldsymbol{R}}$ | **Bulk ESS** | **Tail ESS** |
| --- | --- | --- | --- | --- | --- | --- |
| Fixed Effects | | | | | | |
| Age | 0.10 | 0.25 | -0.42, 0.62 | 1.00 | 4527 | 2966 |
| Random Effects | | | | | | |
| σ_Intercept_ | 0.19 | 0.20 | 0.01, 0.75 | 1.00 | 5819 | 5777 |
| σ_Age_ | 0.40 | 0.31 | 0.02, 1.18 | 1.00 | 3825 | 3441 |
| ρ_Intercept, Age_ | -0.01 | 0.59 | -0.96, 0.96 | 1.00 | 8239 | 10427 |
| Residual Standard Deviation | | | | | | |
| σ | 0.99 | 0.08 | 0.85, 1.16 | 1.00 | 15282 | 13279 |
| R^2^ = 0.07 (95% CrI = 0.01, 0.18) | | | | | | |

*Supplementary Table 4.* Fixed and random effects and residual standard deviation σ of Bayesian linear mixed effects model of association between GABA+/Cr and MoCA, adjusted for age and education.

| **Parameter** | **β** | **Est. Error** | **95% CrI** | $\hat{\boldsymbol{R}}$ | **Bulk ESS** | **Tail ESS** |
| --- | --- | --- | --- | --- | --- | --- |
| Fixed Effects | | | | | | |
| GABA+/Cr | -0.13 | 0.18 | -0.50, 0.23 | 1.00 | 9226 | 7469 |
| Age | -0.05 | 0.24 | -0.54, 0.46 | 1.00 | 9244 | 8134 |
| Education | 0.10 | 0.17 | -0.24, 0.44 | 1.00 | 10416 | 8134 |
| Random Effects | | | | | | |
| σ_Intercept_ | 0.18 | 0.20 | 0.01, 0.71 | 1.00 | 8962 | 8317 |
| σ_GABA+/Cr_ | 0.23 | 0.23 | 0.01, 0.85 | 1.00 | 8072 | 9380 |
| σ_Age_ | 0.40 | 0.28 | 0.03, 1.13 | 1.00 | 7786 | 8871 |
| σ_Education_ | 0.22 | 0.21 | 0.01, 0.79 | 1.00 | 9420 | 9841 |
| ρ_Intercept, GABA+/Cr_ | -0.00 | 0.46 | -0.83, 0.82 | 1.00 | 18673 | 11613 |
| ρ_Intercept, Age_ | -0.00 | 0.46 | -0.82, 0.82 | 1.00 | 15113 | 14477 |
| ρ_GABA+/Cr, Age_ | -0.03 | 0.45 | -0.83, 0.80 | 1.00 | 14012 | 15804 |
| ρ_Intercept, Education_ | 0.00 | 0.45 | -0.82, 0.82 | 1.00 | 21621 | 13324 |
| ρ_GABA+/Cr, Education_ | -0.01 | 0.46 | -0.83, 0.81 | 1.00 | 16648 | 14662 |
| ρ_Age, Education_ | 0.09 | 0.45 | -0.77, 0.85 | 1.00 | 15239 | 15423 |
| Residual Standard Deviation | | | | | | |
| σ | 0.98 | 0.07 | 0.85, 1.14 | 1.00 | 24834 | 13567 |
| R^2^ = 0.13 (95% CrI = 0.04, 0.24) | | | | | | |

Supplementary Figure 1. Posterior predictive plot of Bayesian linear mixed effects model of association between age, gray matter fraction, and GABA+/Cr in oldest-old adults (N=100). The black line represents the observed distribution of scaled GABA+/Cr; blue lines represent 100 draws from the posterior probability distribution.


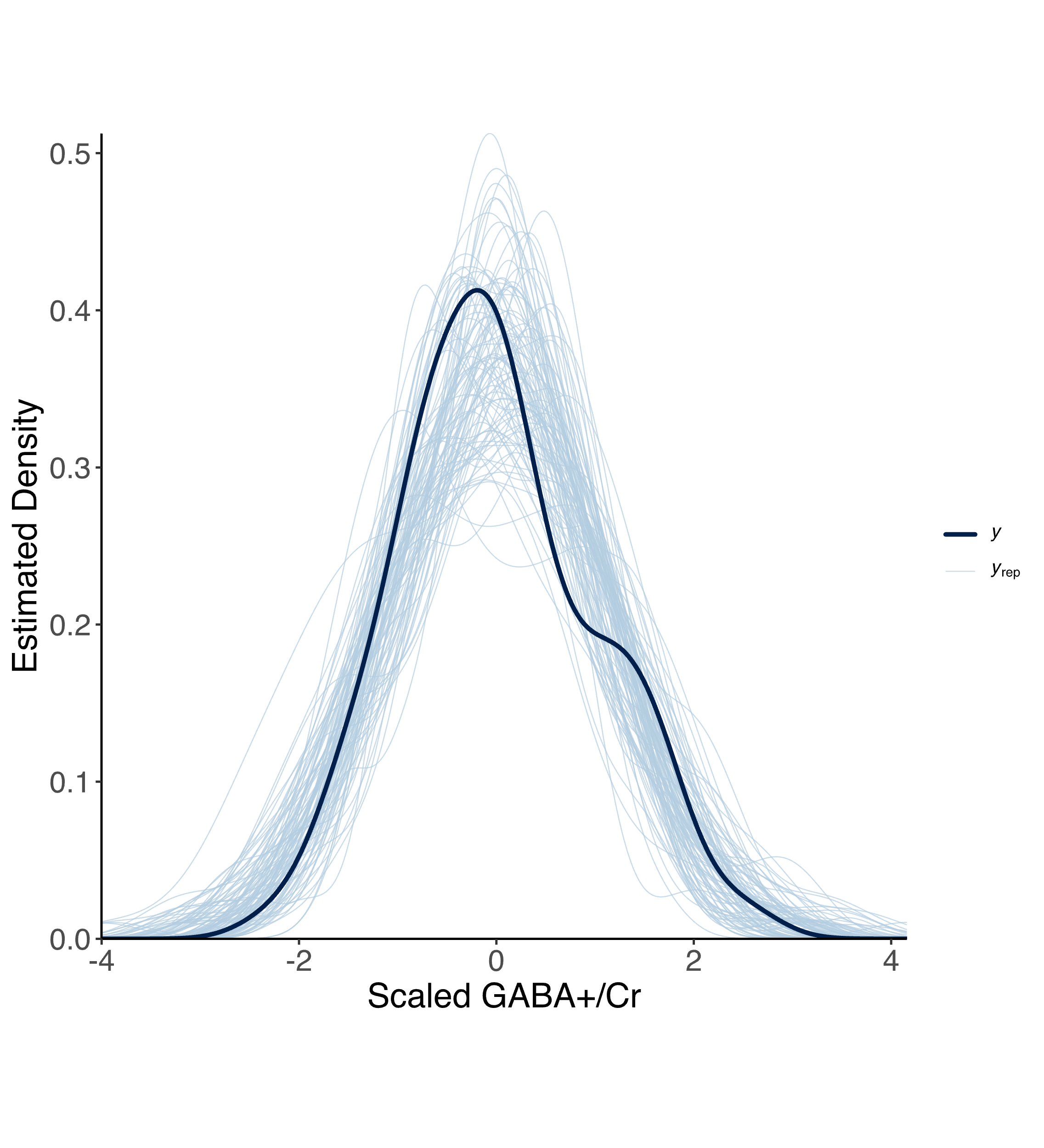


Supplementary Figure 2. Posterior predictive plot of Bayesian linear mixed effects model of association between age, gray matter fraction, age-by-sex interaction, and GABA+/Cr in oldest-old adults (N=100). The black line represents the observed distribution of scaled GABA+/Cr; blue lines represent 100 draws from the posterior probability distribution.


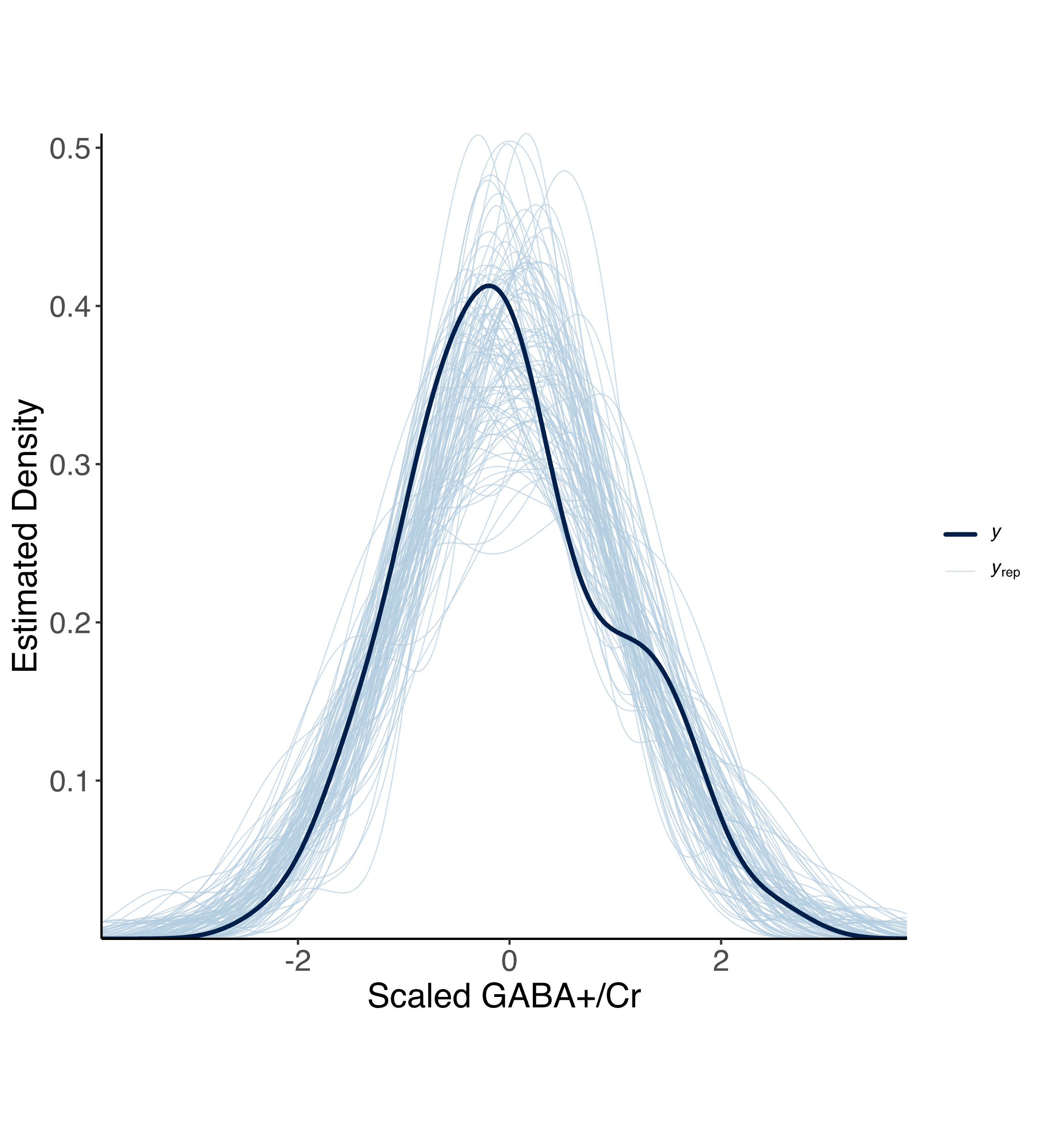


Supplementary Figure 3. Posterior predictive plot of Bayesian linear mixed effects model of association between age, years of education, GABA+/Cr, and MoCA score in oldest-old adults (N=100). The black line represents the observed distribution of scaled total MoCA score; blue lines represent 100 draws from the posterior probability distribution.
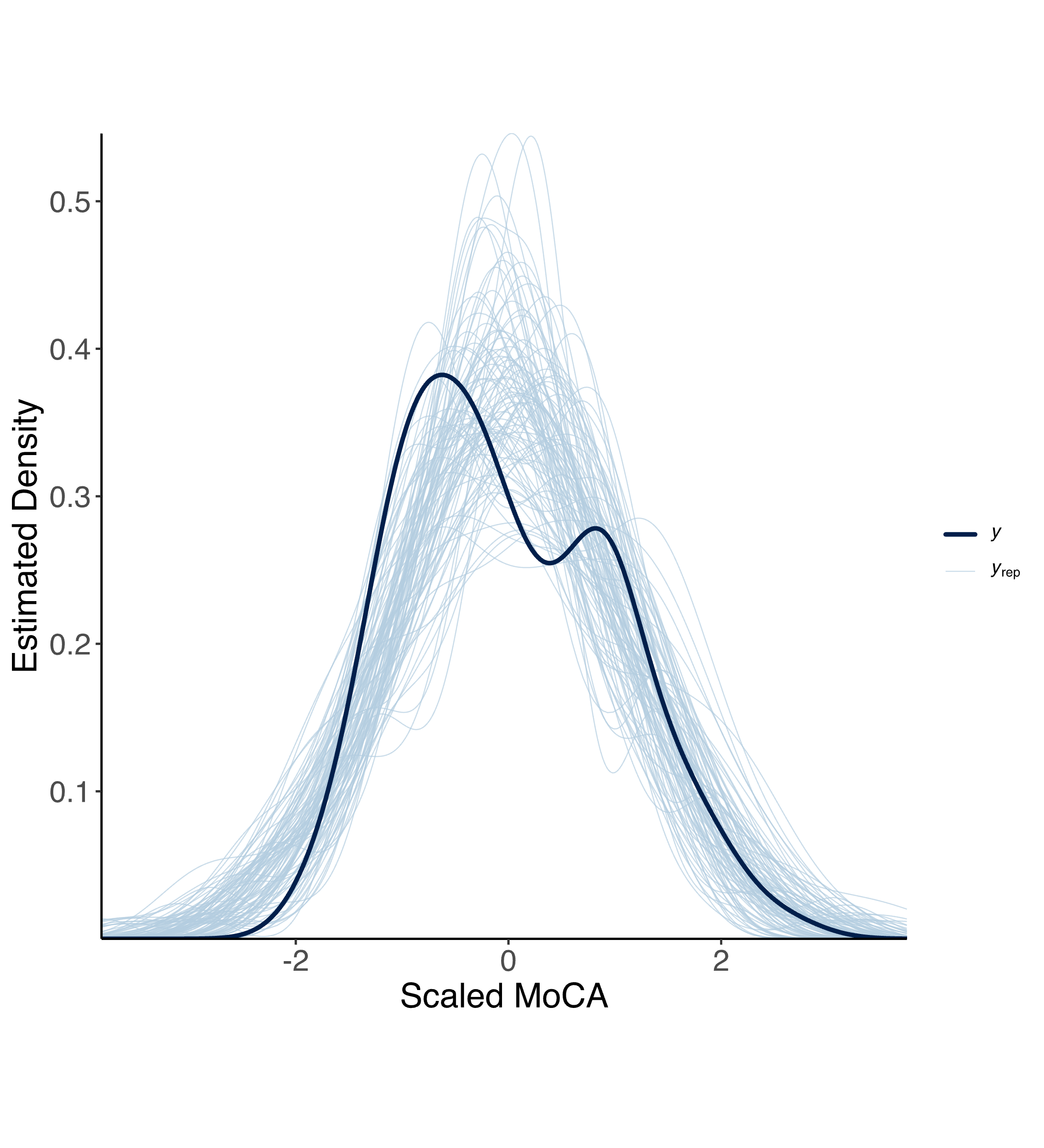


Supplementary References

1. Lin A, Andronesi O, Bogner W, et al. Minimum Reporting Standards for in vivo Magnetic Resonance Spectroscopy (MRSinMRS): Experts’ consensus recommendations. *NMR in Biomedicine*. 2021;34(5):e4484. doi:10.1002/nbm.4484

2. Mikkelsen M, Barker PB, Bhattacharyya PK, et al. Big GABA: Edited MR spectroscopy at 24 research sites. *NeuroImage*. 2017;159:32-45. doi:10.1016/j.neuroimage.2017.07.021

3. Porges EC, Woods AJ, Lamb DG, et al. Impact of tissue correction strategy on GABA-edited MRS findings. *NeuroImage*. 2017;162:249-256. doi:10.1016/j.neuroimage.2017.08.073

1. Data quality metrics derived from a superset of data reported previously^3^ [↑](#footnote-ref-2)
2. Unpublished data quality metrics for these data were provided by the Puts lab. [↑](#footnote-ref-3)
3. Fit indices derived from a superset of data reported previously^3^ [↑](#footnote-ref-4)
